# Influence of Adverse Childhood Experiences and Perfectionism on Musician’s Dystonia: a Case Control Study

**DOI:** 10.5334/tohm.687

**Published:** 2022-03-17

**Authors:** Stine Alpheis, Eckart Altenmüller, Daniel S. Scholz

**Affiliations:** 1 Institute of Music Physiology and Musician’s Medicine, Hannover University of Music, Drama and Media, Hannover, Germany; 2 Department of Education and Psychology, Freie Universität Berlin, Germany

**Keywords:** musician’s dystonia, adverse childhood experiences, emotional neglect, perfectionism, stress resilience

## Abstract

**Background:**

Musician’s dystonia (MD) is a task-specific movement disorder characterized by muscle cramps and impaired voluntary motor-control whilst playing a musical instrument. Recent studies suggest an involvement of adverse childhood experiences (ACEs) in the development of MD.

**Objectives:**

By investigating the prevalence of ACEs in MD patients with perfectionism as possible mediating factor this study aims to gain further insights into the etiology of MD.

**Methods:**

The Adverse Childhood Experiences Scale (ACE-S), the Childhood Trauma Questionnaire (CTQ) and Frost’s Multidimensional Perfectionism Scale (FMPS) were answered by 128 MD patients and 136 healthy musicians. Regression and mediator analyses were conducted to identify relevant predictors of MD and to investigate the role of perfectionism.

**Results:**

The CTQ total score (OR: 1.04; 95% *CI* [1.01, 1.08]) and the sub-score “emotional neglect” (OR: 1.13; 95% *CI* [1.02, 1.25]) were identified as two predictors of MD. Patients scored significantly higher on the sub-score emotional neglect, but no significant differences were observed for other forms of ACEs. Perfectionism had no mediating function on the association between ACEs and MD.

**Discussion:**

Though only slight differences between both groups were found, there is a trend towards higher rates of emotional neglect among dystonic musicians. A possible explanation for the association between musician’s dystonia and emotional neglect could be a lower stress resilience in musicians with a history of ACEs, which increases vulnerability to acquire dysfunctional movement patterns.These tendencies should be further investigated in future studies in which the MD and HM groups are more evenly matched in sex and age.

**Highlights:**

We investigated the role of Adverse Childhood Experiences in the development of musician’s dystonia, comparing a large sample of healthy musicians and dystonia patients. Our findings suggest that experiencing emotional neglect might increase the probability to acquire musician’s dystonia. The findings offer new implications for etiology and treatment of dystonia.

## Introduction

Musician’s dystonia (MD) is a task-specific movement disorder, also known as musician’s cramp. It is characterized by an impairment of voluntary motor control while performing a specific, extensively trained task [1] and manifests through co-contractions of antagonistic muscle groups, leading to involuntary cramps whilst playing a musical instrument [2]. While the disorder is not painful, it is perceived as highly stressful and disabling, often ending a career as professional performer [3].

Recent studies have on the one hand discussed dysfunctional cortical reorganization, a lack of motor inhibition [4], as well as abnormalities in the cerebellum [5,6] and basal ganglia circuitry [7,8] as pathophysiological correlates of MD or similar task-specific dystonias, such as “writer’s cramp”. On the other hand, elevated levels of anxiety, perfectionism and neuroticism [9,10], as well as depression and obsessive compulsive disorder are associated with musician’s dystonia [11]. Known risk factors are furthermore male sex [12,13], a genetic predisposition [14] and a late age when first starting to learn the instrument [15]. Yet the chain of influencing or triggering factors in the etiology of musician’s dystonia remains not fully understood and there is an ongoing discussion as to whether musician’s dystonia is more related to overuse and anxiety or to a genetically and neurophysiologically defined dystonic syndrome [16].

Current studies focus on reports of adverse childhood experiences (ACEs) from patients with musician’s dystonia [17,18]. The term “adverse childhood experiences” includes different forms of abuse, neglect, and household dysfunction, all of which have been found to increase health risk behavior in adults, which subsequently leads to higher rates of ischemic heart disease, cancer, and stroke [19]. All forms of ACEs are furthermore associated with increased prevalence of anxiety disorders, mood disorders, behaviour disorders, substance abuse and personality disorders, as well as psychogenic movement disorders [20]. Additionally, ACEs are believed to foster perfectionism [21] and anxiety [22,23], which are both associated with MD. According to the social reaction model, children might react by showing perfectionistic behavior as a way of coping after traumatic experiences, believing that by acting flawlessly they can regain control and some of the lost affection from the parent [24].

On a neurobiological level, ACEs are known to influence stress networks in adults by affecting the regulation of the hypothalamic-pituitary-adrenal (HPA) axis [25]. As result, stressful situations might be assessed as “threatening” more quickly, leading to a noradrenergic activation of the basolateral amygdala, which promotes emotion-induced consolidation of dysfunctional movement patterns [26,27], thereby affecting movement learning and motor memory [1]. Studies comparing people who had experienced adverse childhood events with those who had not furthermore found alterations in areas including the motor cortex [28], the prefrontal cortex, the cerebellum and the limbic system [29].

Adverse childhood experiences could therefore contribute to the genesis of musician’s dystonia by affecting psychological dispositions, stress regulation as well as sensorimotor networks. Regarding the discussion of the dystonia-classification, an involvement of ACEs in the etiology would support the theory that MD is not only the result of motor circuit dysfunctions of the basal ganglia and the cerebellum, but also a manifestation of dysfunctional stress-coping-mechanisms. Different degrees of involvement of emotional-memory pathways through the limbic system and frontal cortical areas could offer an explanation for the differences in symptom severity and symptom expression observed in MD patients [30].

An involvement of ACEs in the development of musician’s dystonia would further give new implications for individually tailored treatment methods and offer vital information to the etiology discussion of dystonia.

The current study therefore investigates the psychological influence of adverse childhood experiences on MD, the first hypothesis being that ACEs increase the probability of developing dystonia later in life. For the second hypothesis it was examined whether a greater severity of ACEs leads to a more severe form of MD, measured as the subjectively perceived disability reported by the participants. Finally, the third hypothesis states that ACEs may lead to a specific manifestation of perfectionism, which therefore could act as mediator between ACEs and MD.

## Methods

### Participants and Design

The present study followed a case-control design, in which the subjects’ experiences were observed retrospectively. Three hundred and seventy musicians, who had presented themselves at the Institute of Music Physiology and Musicians’ Medicine Hanover (IMMM) between 2004 and 2018 and had received the diagnosis “musician’s dystonia” were contacted with personally addressed letters. Letters from 112 people were returned due to outdated addresses and three patients had died since their last contact with the institute. Of the 255 successfully delivered questionnaires, 121 patients (47.45%) participated by returning their completed questionnaires to the institute. To generate a healthy control group and to reach possibly more musicians with MD, the managements of 18 professional orchestras were contacted with the request to forward the online version of the questionnaires to their musicians, and the web-link was also posted on social media. Of the unknown number of recipients of the web-link, 157 musicians completed the online version of the questionnaire, making it impossible to determine a response rate. To control for a possible response bias, however, childhood trauma and ACEs were not mentioned in the introduction to the questionnaire. Musicians of both, the patient and the control group, were rather invited to contribute to a research project concerning the development of movement disorders in musicians, without giving information about the underlying hypothesis.

From the total of 278 participants, eleven participants were excluded due to other movement disorders besides MD, and three participants were excluded due to too much missing data. Of the 264 remaining participants, 128 musicians (28.13% female) belonged to the MD group and 136 musicians (59.56% female) stated to suffer from no movement disorder, thereby forming a healthy control group (HM).

All participants gave informed consent to participate in the study and received information about the studies complete data confidentiality. Helpful telephone numbers and website-links were provided to offer support, while participants were also encouraged to contact the authors if needed, since the questionnaires concerned possibly troubling topics. The common ethics committee of the Leibniz University and the University of Music, Drama and Media, Hannover approved the study design (ID 197 05 2021).

### Data Assessment

All participants answered questions concerning their demographic data and their medical history of MD. This included the age at the start of playing, the onset of their dystonia and their subjectively perceived disability, which was measured on a 5-point Likert-scale ranging from *no/minimal disability* (1) to *maximum disability/unable to play* (5). For measuring ACEs, the German versions of the Childhood Trauma Questionnaire (CTQ) [31,32] and the Adverse Childhood Experiences Scale (ACE-S) [19,33] were used. The CTQ consists of 28 items, covering emotional, physical and sexual abuse, as well as emotional and physical neglect. Each sub-score consists of five items, while the remaining three items measure the tendency trivialize and deny. All items begin with the phrase “When I was growing up” and are answered on a 5-point Likert-scale, ranging from *never true* (1) to *very often true* (5). The ACE-S consists of ten items that are answered on a dichotomous yes/no scale: (1) emotional, (2) physical and (3) sexual abuse, (4) emotional and (5) physical neglect, (6) separation from a parent, (7) violent treatment towards mother, (8) substance abuse, (9) mental illness or (10) imprisonment of a household member. The sum of all yes-answers forms the so-called ACE score. The scale was extended with an additional item (“ACE11”) for the present study, asking after other events during childhood and adolescence that had been perceived as traumatic or highly stressful.

Frost’s Multidimensional Perfectionism Scale (FMPS) was used for measuring perfectionism [34,35]. The scale consists of 35 items measuring four dimensions of perfectionism on a 5-point Likert-scale, ranging from *strongly disagree* (1) to *strongly agree* (5): concern over mistakes and doubts (CMD), parental expectations and criticism (PEC), personal standards (PS) and organization (O). The corresponding items are added up to form each sub-score. To calculate an overall perfectionism-score, all sub-scores are summed up, except for the score of organization.

### Statistical Analysis

All statistical analyses were conducted in RStudio, R version 3.4.2 [36]. All demographic data and questionnaires (CTQ, ACE-S, FMPS) were examined regarding their distribution and descriptive analyses were conducted to portray mean and standard deviation, as well as median and interquartile ranges for all non-parametric data. The Wilcoxon rank-sum test was applied to compare demographic data, and the total and sub-scores of the questionnaires for both groups, while a chi-square test was performed to compare sex distributions between the groups. The frequency of having experienced an additional traumatic event (ACE11) was compared separately, and the subject’s answers were analyzed qualitatively, using thematic data analysis.

All variables were *z*-standardized before regression analyses and correlated to investigate possible associations. Multiple logistic regression with the CTQ total score as predictor and disorder (MD vs. HM) as criterion was applied to examine the role of ACEs in the development of musician’s dystonia (Model 1a). To control for possible influences from other factors, the association of demographic variables with the CTQ total score was analyzed with multiple linear regression beforehand. Age at the start of playing and sex which are known influencing factors on MD were included into an adjusted logistic regression model (Model 1b) as covariates. The participant’s current age, however, was deliberately not included into the adjusted models, since the age at the time of filling in the questionnaire was not relevant for the current hypotheses as previous studies have not found an age-related recall bias when measuring self-reported childhood trauma [37]. To further investigate the influence of the CTQ sub-scores on musician’s dystonia, all sub-scores were entered as predictors in a new uncontrolled model (Model 2a), as well as in a model adjusted for age at the start of playing and the participant’s sex (Model 2b). Using further logistic regression models, the influence of the ACE score on musician’s dystonia was investigated in the same manner.

To assess whether adverse childhood experiences are a predictor for higher subjective motor disability due to MD, ordinal logistic regression was performed, including subjective disability as criterion and the same predictors as in the previous analyses. Contrary to the analysis of the first hypothesis, current age was included into the ordinal regression model of the second hypothesis as covariate, since a correlation between age and subjective disability was observed in the MD group and it is possible that the current subjective disability depends on the current age.

Since all healthy musicians reported to suffer from no movement disorder, the value “1” was inserted to reflect no subjective motor disability for all participants of the HM group. In this step, it was further analyzed how perfectionism influences the subjective disability by including the FMPS score into ordinal regression as a predictor and as an interaction variable.

Lastly, to test whether perfectionism acts as mediator between ACEs and MD, a mediation analysis was performed.

To verify there was no violation of the assumption of linearity of the logit, all variables were tested before regression analyses, and the absence of multicollinearity in the models was tested through the variance inflation factor.

## Results

### Participants Characteristics and Descriptive Results

Descriptive analyses (***Table 1***) revealed a significant difference in the average ages, and sex distributions of both groups and showed that MD patients had also started to learn their instruments significantly later.

**Table 1:** Descriptive characteristics and questionnaire scores of both groups.


PARAMETER	MD (*n* = 128)	HM (*n* = 136)	TEST STATISTIC	*p*-VALUE	ADJUSTED *p*-VALUE

	*MDN (IQR)*	*MDN (IQR)*			

Age in years (*M* ± *SD*)	56.13 (10.63)	38.03 (12.27)	14795	.000***	

Sex: male/female/other (*n*)	92/36/0	55/81/0	χ^2^(1, *N* = 264) = 25.14	.000***	

Age started playing the instrument	9.5 (5)	7.0 (4)	12314	.000***	

Current occupation: professional/student/other/NA (*n*)	117/2/5/4	106/30/0/0			

Dystonia onset: age (*M* ± *SD*)	38.46 (11.73)	–			

Subjective motor disability at instrument (*M* ± *SD*)	3.66 (1.19)	–			

Subjective motor disability in everyday life (*M* ± *SD*)	1.57 (0.90)	–			

CTQ					

Total Score	33.50 (12.25)	32 (8)	10292	.01**	.052

Emotional abuse	6 (4)	6.5 (3)	8834.5	.83	.830

Physical abuse	5 (1)	5 (0)	9730	.029*	.110

Sexual abuse	5 (0)	5 (0)	9521	.028*	.110

Emotional neglect	10 (6)	8 (5)	11046	.000***	.000***

Physical neglect	6 (3)	5 (2)	9740.5	.077	.154

ACE score	0 (0)	0 (0)	8849	.799	1

FMPS					

Total Score (without O)	78 (34.25)	82 (28)	8336.5	.554	1

Concerns about Mistakes and Doubts	38 (19)	38 (16.25)	8525	.773	1

Parental Expectations and Criticism	20 (12.25)	20 (12)	8489	.729	1

Personal Standards	23.5 (10)	24 (8)	8271	.485	1

Organization	23 (6)	24 (5)	8200.5	.415	1


*Note*: Subjective motor disability measured on a 5-point Likert-scale ranging from 1 (*no/minimal disability*) to 5 (*maximum disability/unable to play*). Median and interquartile ranges are displayed, unless otherwise indicated. Test statistics show *W* of the Wilcoxon rank-sum test unless otherwise indicated. *p*-values for CTQ, ACE and FMPS scales were Holm-corrected. *** *p* < .001; ** *p* < .01; * *p* < .05. Abbreviations: MD = musician’s dystonia patients, HM = healthy musicians, CTQ = Childhood Trauma Questionnaire, ACE = Adverse Childhood Experiences, FMPS = Frost’s Multidimensional Perfectionism Scale, O = Organization.

**Figure 1:**
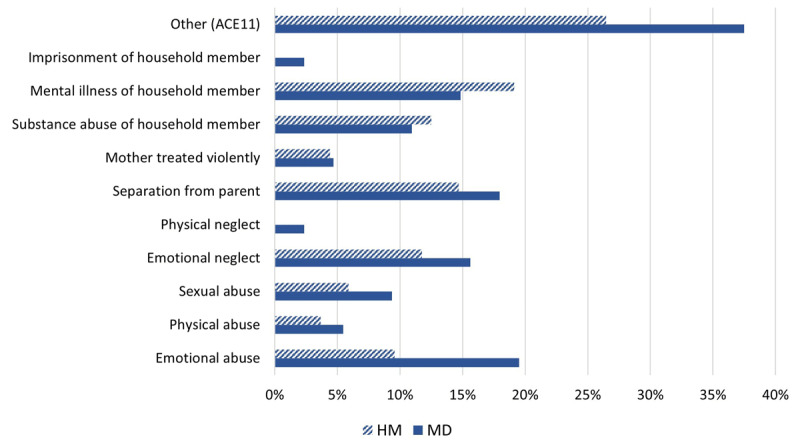
**Reported frequencies of different forms of Adverse Childhood Experiences on the ACE-scale.** Percentages of „yes“-answers per group. * *p* < .05. Abbreviations: ACE = Adverse Childhood Experiences; HM = healthy musicians; MD = musician’s dystonia patients.

Descriptive analysis further revealed higher rates of emotional neglect and a trend towards a higher CTQ total score for MD patients. The average emotional neglect score of the MD group can be classified as *slight to moderate* (classification by Häuser et al. [38]), while it was *none to minimal* among the healthy controls. The Wilcoxon rank-sum test showed only the difference on the sub-score emotional neglect (EN) to be significant after Holm correction, the effect for the difference being small (*r* = .24, 95% *CI* [0.11, 0.35]). Therefore, apart from the CTQ total score, the sub-score “emotional neglect” was specially observed in further analyses. No significant differences were observed between the median of the ACE scores of both groups (***Table 1***), but descriptive analysis showed more frequent occurrences of almost all forms of ACEs among the MD group (***Figure 1***).

Especially “emotional abuse” was reported more often by the MD group, but this difference was not significant after Holm-correction (χ^2^(1, *N* = 264) = 4.5, *p* = .33). The item ACE11 (“other stressful experiences”) was reported by 37.5% (*n* = 48) of the MD patients and by 26.5% (*n* = 36) of the healthy musicians. Using qualitative thematic analysis, 15 themes were identified from the musician’s written answers to ACE11 (***Table 2***). Most prominent among the MD group were further reports of emotional distance or neglect, conflicts among family members that were partly violent, emotional abuse from family members, and performance pressure at the instrument. Themes most often reported by the HM group were bullying at school and non-violent conflicts among family members.

**Table 2:** Qualitative thematic analysis of “other stressful experiences”.


IDENTIFIED THEMES	ABSOLUTE NUMBERS OF REPORTS (%)	

	HM (*n* = 136)	MD (*n* = 128)

Emotional distance/neglect	2 (1,5)	11 (8.6)

Emotional and psychological abuse	1 (0.7)	6 (4.9)

Conflicts among family members	7 (0.7)	7 (5.5)

Victim or witness of physical violence	2 (1,5)	5 (3.9)

Separation from parent(s)/Death of a parent, family member or close friend	3 (2.2)	6 (4.9)

(Assumed) sexual abuse	1 (0.7)	4 (3.1)

Mental illness in childhood	3 (2.2)	6 (4.9)

Physical illness in childhood	–	2 (1.6)

Mental disorder of a parent	1 (0.7)	5 (3.9)

Alcohol abuse of a parent	1 (0.7)	5 (3.9)

Divorce/separation of parents	2 (1,5)	2 (1.6)

Bullying at school/in the neighbourhood	8 (5.9)	2 (1.6)

Hospital stay alone	2 (1,5)	1 (0.8)

Performance pressure, negative experiences at the instrument	4 (2.9)	6 (4.9)

Other	2 (1,5)	3 (2.5)

**Total number of reported events**	**39**	**71**


*Note*: Themes were identified according to the written answers participants gave to the question “Were there any other events in your childhood or adolescence that you would rate as extremely stressful?”

There were no significant differences between MD and HM in the total score of Frost’s Multidimensional Perfectionism Scale and its sub-scores, with all *p*-values equaling *p* = 1 after Holm correction.

**Figure 2:**
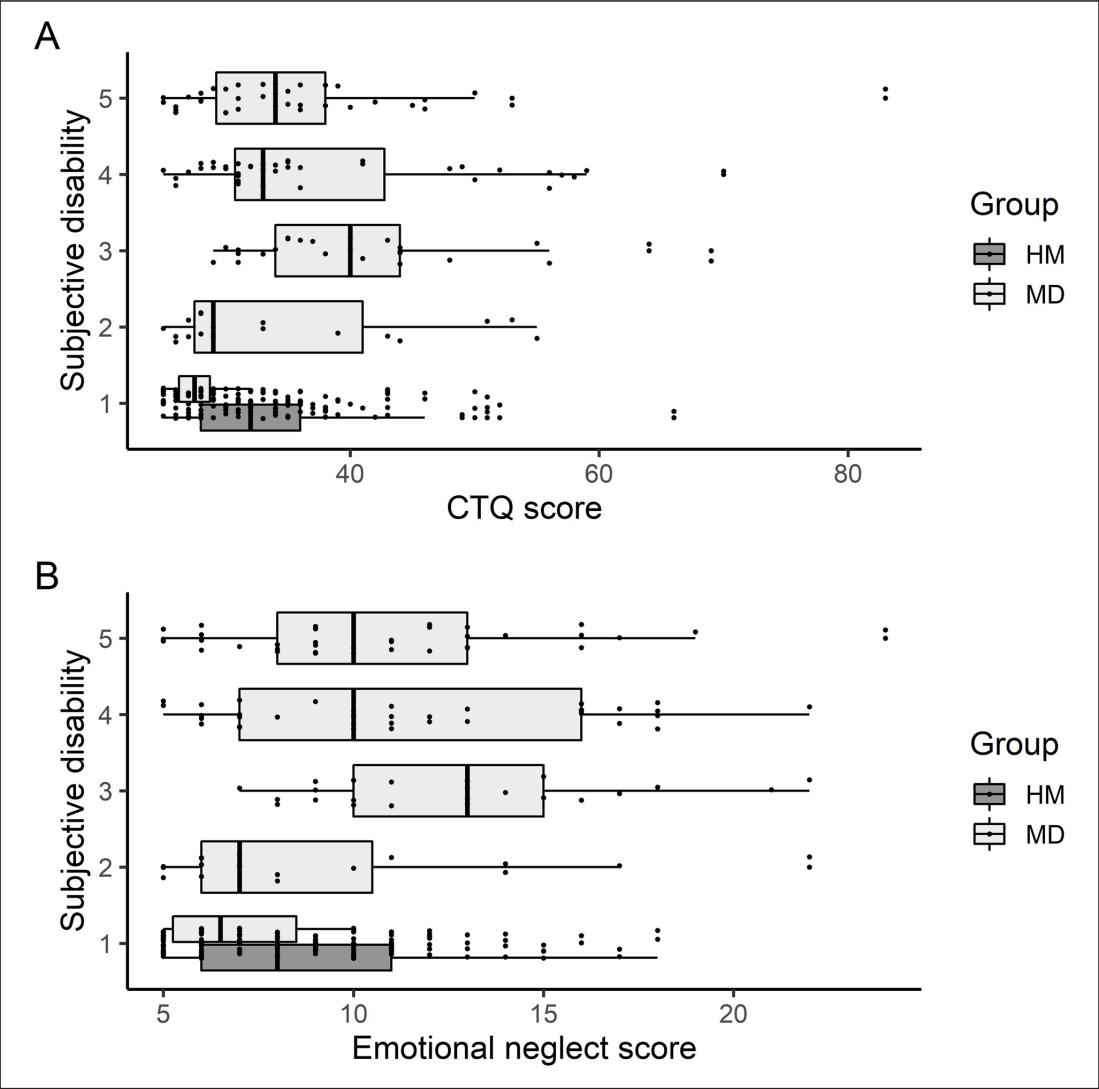
**Distribution of CTQ total (A) and emotional neglect (B) scores among the ratings of subjective disability.** 1 = *no/minimal disability*; 5 = *maximum disability/unable to play*. Abbreviations: CTQ = Childhood Trauma Questionnaire; EN = emotional neglect.

Regarding subjective motor disability at the instrument, descriptive analysis revealed slightly higher CTQ and emotional neglect scores for those participants with a subjectively more severe motor disability, but patients who rated their disability to be *moderate* (3) on average scored the highest total score on the CTQ (*Mdn(IQR*) = 40(10.25)) and on the sub-score emotional neglect (*Mdn(IQR*) = 13(5.25)) (***Figure 2***).

Spearman’s rank correlation revealed the CTQ total score and EN to be amongst others positively correlated with age, disorder, subjective disability and the FMPS score (***Table 3***).

**Table 3:** Bivariate correlation (Spearman’s rho) of relevant demographic, childhood trauma and perfectionism variables.


	GENDER	AGE	DISORDER	SUBJECTIVE DISABILITY	START OF PLAYING	CTQ SCORE	EMOTIONAL NEGLECT	ACE SCORE	FMPS SCORE

Gender	–								

Age	–.34***	–							

Disorder	–.33***	.60***	–						

Subjective disability	–.30***	.60***	.90***	–					

Start of playing (age)	–.26***	.29***	.36***	.33***	–				

CTQ score	–.02	.16*	.15*	.18**	.10	–			

Emotional neglect	–.09	.23***	.23***	.26***	.17**	.87***	–		

ACE score	.06	.07	.03	.03	.06	.58***	.43***	–	

FMPS score	.24***	–.09	–.03	.00	–.17*	.36***	.25***	.32***	–


*Note*: Reference categories: gender (0 = male; 1 = female); disorder (0 = no movement disorder, 1 = musician’s dystonia). *** *p* < .001; ** *p* < .01; * *p* < .05. Abbreviations: CTQ = Childhood Trauma Questionnaire, ACE = Adverse Childhood Experiences, FMPS = Frost’s Multidimensional Perfectionism Scale.

### Logistic Regression

Linear regression revealed significant associations between CTQ total score and age (*β(SE)* = .10 (.04), *t*(260) = 2.46, *p* < .05), as well as CTQ total score and age at the start of playing ((*β(SE)* = .43 (.17), *t*(260) = 2.46, *p* < .05). The association between age and CTQ total score, however, was only observable for the total sample. When both groups (MD and HM) were analyzed separately, the CTQ total score was not associated with the age of the participants, which gives additional support to the decision not to include age as covariate, as described in the methods section.

The participant’s sex was not found to be associated with ACEs.

In the restricted logistic regression model with only the CTQ total score as predictor (Model 1a), the CTQ total score was found to significantly contribute to the prediction of MD and the model was found to be a significant improvement to the baseline model (see ***Table 4***). The explanatory value of the CTQ total score remained significant when sex, and age at the start of playing were additionally included into the model (Model 1b). Compared to a model with only sex and age at the start of playing as predictors, including the CTQ total score into the model contributed significantly to an improvement of its explanatory value (χ^2^(*df*) = 7.03(2), *p* < .01). Model 1b can be viewed as acceptable according to the calculated pseudo *R^2^* = .26.

**Table 4:** Predictors of musician’s dystonia in uncontrolled and controlled logistic regression models.


VARIABLES	MODEL 1A				MODEL 1B			
	
	*β (SE)*	OR	95% *CI* FOR ODDS RATIO		*β (SE)*	OR	95% *CI* FOR ODDS RATIO	
	
			LL	UL			LL	UL

Constant	–0.05 (0.13)				–0.66 (0.21)**			

CTQ Score	0.05 (0.01)**	1.05	1.02	1.08	0.04 (0.02)*	1.04	1.01	1.08

Age at start of playing					0.20 (0.05)***	1.23	1.12	1.35

Sex					1.13 (0.28)***	3.10	1.80	5.43

χ^2^ (*d*f)	10.73 (1)**				57.95 (3)***			

Nagelkerke *R^2^*	.05				.26			

	**MODEL 2A**				**MODEL 2B**			

Constant	–0.05 (0.13)				–0.68 (0.22)**			

Emotional abuse	–0.10 (0.06)	0.91	0.80	1.02	–0.02 (0.07)	0.98	0.86	1.12

Physical abuse	0.12 (0.10)	1.12	0.92	1.39	–0.00 (0.12)	1.00	0.78	1.26

Sexual abuse	0.04 (0.08)	1.04	0.90	1.24	0.12 (0.09)	1.13	0.96	1.38

Emotional neglect	0.17 (0.05)***	1.19	1.09	1.30	0.12 (0.05)*	1.13	1.02	1.25

Physical neglect	–0.04 (0.08)	0.96	0.82	1.11	–0.07 (0.08)	0.94	0.80	1.10

Age at start of playing					0.20 (0.05)***	1.22	1.11	1.25

Sex					1.16 (0.30)***	3.20	1.79	5.81

χ^2^ (*df*)	21.32 (5)***				62.66 (7)***			

Nagelkerke *R^2^*	.10				.28			


*Note*: Model 1a and 2a: crude associations. Model 1b and 2b: adjusted for sex and age at the start of playing. χ^2^ statistics show relation of explanatory value of the model compared to the baseline model. *** *p* < .001; ** *p* < .01; * *p* < .05. Abbreviations: CI = confidence interval; CTQ = Childhood Trauma Questionnaire; LL = lower level; OR = odds ratio; UL = upper level.

In a closer examination of the CTQ sub-scores (***Table 4***), emotional neglect was identified as the single significant predictor for musician’s dystonia of all sub-scores in an uncontrolled model (Model 2a), as well as in a model additionally including sex and age at the start of playing. The calculated pseudo *R^2^* =.28 for the controlled model (Model 2b) again showed an acceptable fit of the model.

In a separate regression model, the ACE score was not found to have a significant explanatory value for musician’s dystonia in musicians (*β* = 0.13, *SE* = 0.09, Model χ^2^(*df*) = 2.12(1), *p* = .15), and neither was the FMPS score (*β* = –0.00, *SE* = 0.01, Model χ^2^(*df*) = 0.09(1), *p* = .76).

### Ordinal Regression

In the ordinal logistic regression model uncontrolled for covariates, the CTQ total score was found to be a significant predictor for a higher rating of subjective disability (*β* = 0.04, *SE* = 0.01, *Wald* = 3.14, *p* < .01) and the same held true for emotional neglect (*β* = 0.12, *SE* = 0.03, *Wald* = 4.25, *p* < .001). The odds ratio for rating subjective disability one category higher, with a higher CTQ total score of one unit, was 1.04 (95% *CI* [1.01, 1.06]), while it was 1.13 (95% *CI* [1.07, 1.19]) for a higher score of one unit on the scale emotional neglect.

**Figure 3:**
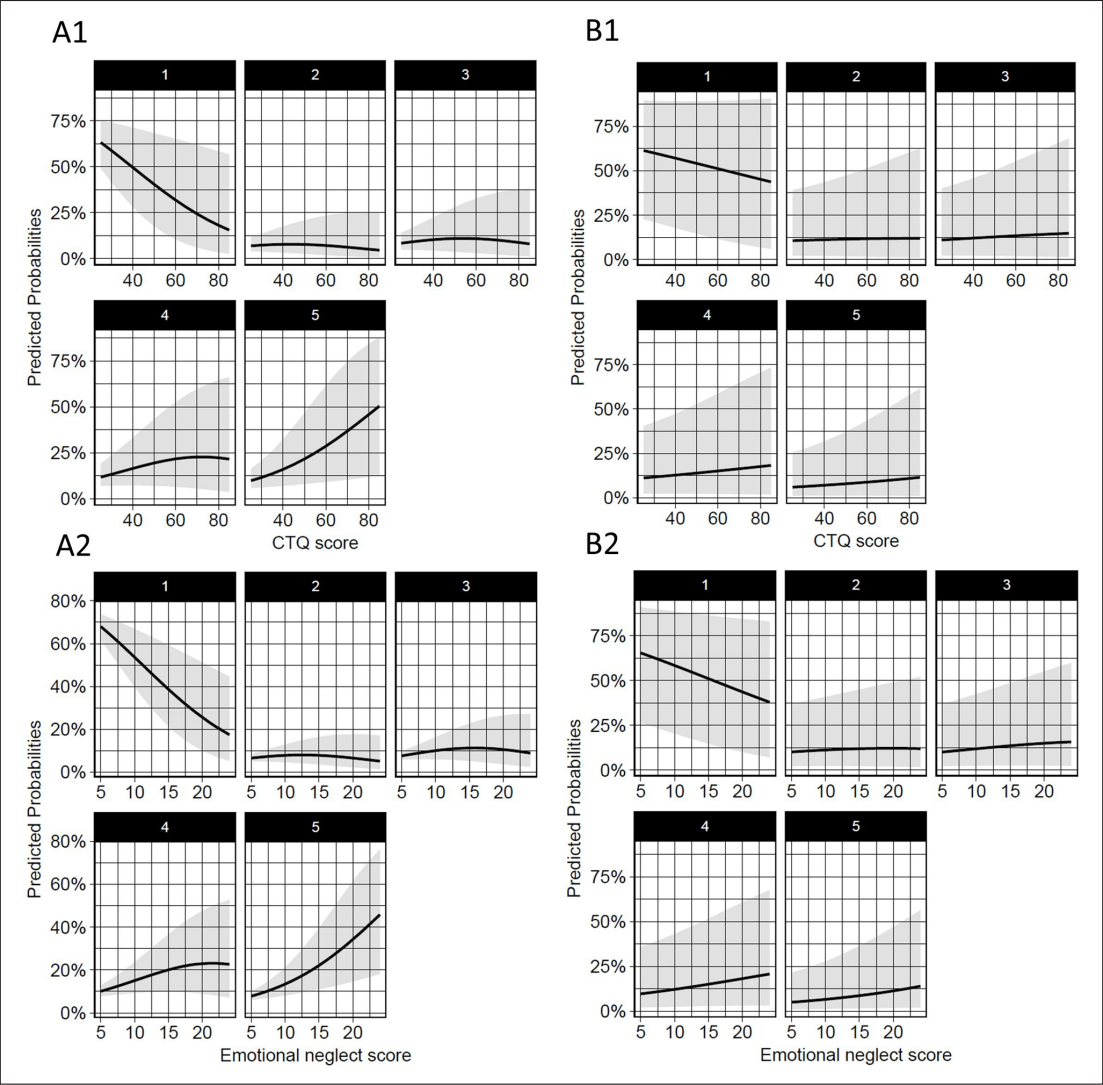
**Predicted probabilities of experiencing a certain degree of subjective disability.** 1 = *no/minimal disability*, 5 = *maximum disability/unable to play*, based on CTQ total and emotional neglect scores in an uncontrolled ordinal regression model (A1 & A2) and in a model adjusted for age, sex, and age at the start of playing (B1 & B2).

However, in an adjusted model including the CTQ total score, age, sex, and age at the start of playing, current age (*β* = 0.10, *SE* = 0.01, *Wald* = 8.12, *p* < .001) and age at the start of playing (*β* = 0.09, *SE* = 0.04, *Wald* = 2.13, *p* < .05) were found to be the only predictors contributing to higher ratings of subjective motor disability (for graphic analysis, see ***Figure 3***).

The ACE score again had no influence on the probability to experience a certain severity of subjective disability (*β* = 0.09, *SE* = 0.08, *Wald* = 1.12, *p* = .26). The FMPS score showed to have no influence on subjective motor disability alone (*β* = 0.00, *SE* = 0.01, *Wald* = 0.21, *p* = .84) and no interaction of the FMPS score with the CTQ total score was found (*β* = 0.00, *SE* = 0.01, *Wald* = 1.61, *p* = .11).

### Mediator Analysis

**Figure 4:**
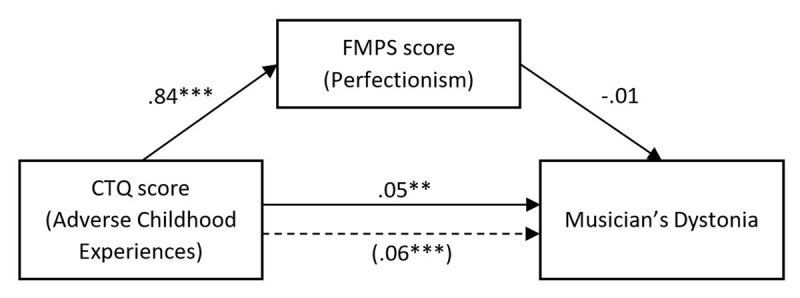
**Standardized regression coefficients for the relationship between CTQ total score and musician’s dystonia as mediated by perfectionism.** In parentheses is the standardized regression coefficient between CTQ total score and musician’s dystonia, controlling for perfectionism. *** *p* < .001, ** *p* < .01. Abbreviations: CTQ = Childhood Trauma Questionnaire; FMPS = Frost’s Multidimensional Perfectionism Scale.

No mediating effects of the FMPS score between the CTQ total score and musician’s dystonia were observed. While there was a significant association between CTQ total score and FMPS score with the CTQ explaining 14% of the variance in perfectionism (*R^2^* = .14, *F*(1,262) = 44.26, *p* < .001), the FMPS score had no significant effect on musician’s dystonia (see ***Figure 4***).

The indirect effect from CTQ on musician’s dystonia through perfectionism was –0.00 (95% *CI* [–0.00, 0.00]), showing no increase of probability. The significance of the indirect effect was tested using a bootstrap procedure [39], resampling the indirect effects 1,000 times, which revealed the indirect effect to be not significant (*p* = .10).

## Discussion

The present study compared dystonia patients with healthy musicians regarding adverse childhood experiences and perfectionism and investigated how these influence the development and subjective severity of musician’s dystonia. MD patients reported significantly higher rates of exposure to emotional neglect in childhood and emotional neglect was found to be a significant predictor for musician’s dystonia, along with the total score of the childhood trauma questionnaire. Other forms of adverse childhood experiences were not associated with musician’s dystonia and neither was perfectionism.

Male sex and a higher age when first learning the instrument also increased the probability for musician’s dystonia, which corroborates previously made findings [15,40].

### ACEs as Risk Factor for Musician’s Dystonia

A broad range of childhood adversities was investigated to test the first hypothesis that ACEs increase the probability of developing MD later in life. While there was a statistically significant association of emotional neglect with musician’s dystonia, this association was not strong and although there were significant differences on the emotional neglect score between the groups, the average severity of emotional neglect reported by the MD patients was merely *slight to moderate*. Additionally, the association might be confounded by the substantial age and sex differences between the groups. However, the combined quantitative and qualitative analyses of the CTQ and ACE-Scale show tendencies towards higher rates of emotional neglect and abuse, as well as performance pressure and fear of rejection in the written reports (item “ACE11”) of the MD patients. The individually written answers to the open item ACE11 and personal notes addressed to the authors by some patients lead to suggest that there is at least a sub-group of patients highly affected by adverse childhood experiences. Due to their individual and subjective nature, however, not all of these adverse experiences seem to fall under the definition of childhood trauma used for the CTQ and the ACE-scale and they are therefore possibly not registered statistically. Studies have further shown that subjectively judged adverse experiences, such as emotional neglect, are overemphazised under conditions of poorer general well-being [41,42], which could be the case for those patients who are especially affected by their movement disorder. Since neither well-being nor depression or anxiety data were collected, a bias in the retrospective reports of those patients suffering from these conditions can not be ruled out. Moreover, some studies suggest that musicians and performing artists in general have experienced more ACES than the non-artistic population, since they are associated with stronger creative and flow experiences, which could be beneficial for professional success [43]. Simultaneously, victims of childhood adversities might turn to music as a resource and a means of escapism.

Nonetheless, it is to be considered that the reported adverse experiences could alter the HPA activity in MD patients, as well as the functional connectivity of sensorimotor networks. We would suggest that those patients with a history of negative childhood experiences are especially vulnerable to stress and that practicing or performing under high pressure could lead to enhanced memory consolidation of possibly dysfunctional motor movements among these musicians [27], thereby promoting the development of musician’s dystonia.

The results offer further suggestions that there are psychological and environmental components in the etiology of musician’s dystonia, and that a broader range of childhood experiences (e.g. the relationships with primary caregivers and instrumental teachers) might play a role that needs to be further investigated. To counterbalance the effects of stress on motor learning, strengthening resilience could be a key component in dystonia treatment, therapy, and prevention.

### ACEs and Subjective Disability

While subjective disability is on the one hand a measure of the individual motor disability musicians observe for themselves, it can also be interpreted as an indicator for the actual severity of musician’s dystonia, which is otherwise only to be measured with objective individual video analysis. Previous studies have shown that there is a correlation between subjectively perceived motor impairments and objectively measured motor performance in musicians [44].

Even though more severe adverse childhood experiences were found to significantly predict the probability of experiencing a higher level of subjective disability in an unadjusted model, the effect vanished when age was included into the model as covariate. Both models need to be interpreted with caution, however, because of the large age difference between the groups. It is likely that the higher number of older patients in the MD group suffers from greater subjective disability than the younger musicians of the healthy control group, not only because of their disorder but also because of age related decline in subjective motor performance [45]. Interestingly, it was observed that patients rating their disability as *moderate* (3), seemed to have experienced severer adverse events and more emotional neglect, while the CTQ total scores for patients with an *extreme* (5) subjective disability were only slightly higher than those of participants with *no or minimal disability* (1). While these findings were not significant, they could support the theory that milder forms of MD are influenced more by psychological factors and stressors, and could be categorized as (at least partly) psychogenic, while more severe forms of musician’s dystonia are less influenced by individual stressors and result rather more from organic dysfunction [46,47].

### Perfectionism

Another surprising finding in the current study was the lack of differences in perfectionistic tendencies between MD patients and healthy controls. Perfectionism in musicians has previously been reported in several studies and is thought to be an important psychological influencing factor of musician’s dystonia [9,30]. There were significant correlations between perfectionism and the severity of adverse childhood experiences, but contrary to the third hypothesis, the influence of ACEs on the severity of musician’s dystonia did not depend on perfectionism, and perfectionism did not contribute to predicting musician’s dystonia. It is possible that MD patients might have tried to adjust their perfectionistic behavior in the time since the diagnosis, or they simply gave their answers in the present study according to a social desirability bias. Other explanations could be that perfectionism is not as relevant in the development of musician’s dystonia as previously assumed or that the similar perfectionism scores reflect the personality characteristics of individuals most likely to participate in voluntary studies, thereby being the result of a response bias [48]. A comparison of perfectionistic tendencies between dystonia patients and healthy controls should therefore be conducted with a naïve sample of musicians.

### Limitations and Outlook

There are limitations to this study that need to be considered for the interpretation of the results. Even though the current age of the participants can be argued to be irrelevant for the studies main question, the large age difference of on average 18 years between the MD patients and healthy controls is a clear limitation. This is partly to be explained by the administration of the questionnaires (personally addressed letters to patients vs. online acquisition of healthy controls). Since the average age of the control group was 38.03 years, the possibility that dystonia had simply not yet manifested in some musicians of the control group needs to be considered, the average age of onset for MD being 35.7 years [49]. The age difference between the groups might further have influenced the reports of adverse childhood experiences, although prior studies have found contradictory results regarding this aspect, with some studies reporting higher prevalences of ACEs in younger age groups [50], while others report higher prevalences in older participants [51]. Additionally, the groups are not matched by sex and even though the sex was not found to be associated with adverse childhood experiences in the current sample, previous studies have described sex differences in retrospective reports of childhood trauma [51] and this could be a confounding factor in the analysis.

The musicians of the control group were not subjected to a medical examination, therefore the absence of movement disorders relies on self-report only, which could be a source for possible misclassification of participants. It should be considered that as a result of dystonia, livelihood and job perspectives of MD patients might be altered, which would possibly influence their current well-being and their retrospective view on childhood experiences. In this case, a control group with musicians who lost their ability to play due to orthopedic reasons or other injuries might have offered a more valid comparison. However, a study by Lee and colleagues found no differences in life-satisfaction and well-being between healthy musicians and dystonia patients [52]. Furthermore, many of the participating patients continue their work as musicians after receiving treatment.

Another limitation is that measuring ACEs comes with methodological challenges, since all statements are made retrospectively in self-administered questionnaires, and childhood maltreatment is subjective and thereby difficult to quantify. Previous studies have shown that adults are sometimes unable to recall early childhood memories correctly or that their memories are influenced by their current well-being, as already discussed above. This can result in artificial links between childhood adversities and adult health outcomes [42,41]. On the other hand, self-administered trauma questionnaires sometimes underly a bias due to social desirability or shame, which leads to under-reporting of certain events. However, collecting prospective data of childhood abuse requires a longitudinal design, which was not possible for the current study.

The highly individual reports of ACEs given by some participants suggest that there are negative childhood events that do not fall under the categories of the applied questionnaires. The reports suggest that instead of a dose-response-relationship between childhood trauma and MD, a single negative event may already shape psychological dispositions and stress vulnerability, thereby affecting movement learning at the instrument. Semi-standardized interviews could help to better objectify the severity of a broader range of ACEs on an individual level. The comparison might also have been influenced by data acquisition bias. MD patients had a personal connection to the IMMM, having been treated there at least once, which might have made them more open to answer questions regarding adverse childhood events. It has further been shown in previous research that patients with mental disorders and under psychological strain are more likely to recall traumatic events, which could hold true for MD patients as well [53,54]. Since the current sample was not screened for the presence of depression or anxiety, it is not possible to determine whether these disorders that are constantly associated with musician’s dystonia had an impact on the reports made by the MD patients. Especially in the light of the above mentioned recall bias of retrospective studies, this is a further limitation for the interpretation of the results that needs to be addressed in future research.

In conclusion, a comparison of adverse childhood experiences between musician’s dystonia patients and healthy musicians showed significant differences only on the sub-score emotional neglect, which therefore possibly plays a role in the development of musician’s dystonia. Though several individual qualitative reports made by the MD patients support this hypothesis, it needs to be considered that the statistical effects for the differences were not strong and might have been confounded by substantial age and sex differences between the groups, as well as by recall bias due to the study design. More severe ACEs were furthermore associated with moderate subjective disability, which could support the hypothesis that a milder form of musician’s dystonia is especially affected by psychological factors. Individual qualitative reports of additional adverse childhood experiences lead to the suggestion that early life stress lowers the levels of resilience and thereby increases stress vulnerability and the manifestation of dysfunctional movements in dystonia patients. Contrary to the third hypothesis, perfectionism was not found to be relevant in predicting musician’s dystonia and played no role as mediator between ACEs and musician’s dystonia.

A follow-up study with groups that are more evenly matched by age, sex, and instrument is already in preparation. Participants will be subjected to a proper medical examination and further psychological data including a broader range of negative childhood events will be assessed. Additionally, neurobiological and neural correlates of ACEs in musicians will be examined to further investigate the association between ACEs and musician’s dystonia.

## Data accessibility statement

The datasets used and/or analysed during the current study are available from the corresponding author on reasonable request.
